# Spintronic terahertz metasurface emission characterized by scanning near-field nanoscopy

**DOI:** 10.1515/nanoph-2023-0858

**Published:** 2024-03-13

**Authors:** Mingcong Dai, Jiahua Cai, Zejun Ren, Mingxuan Zhang, Jiaqi Wang, Hongting Xiong, Yihang Ma, Youwei Wang, Sitong Zhou, Kuiju Li, Zhentao Lv, Xiaojun Wu

**Affiliations:** School of Electronic and Information Engineering, 12633 Beihang University, Beijing, China; School of Electronic and Information Engineering, and School of Cyber Science and Technology, 12633 Beihang University, Beijing, China; Zhangjiang Laboratory, 100 Haike Road, Shanghai, China; Wuhan National Laboratory for Optoelectronics, Huazhong University of Science and Technology, Wuhan, China

**Keywords:** spintronic THz emission, metasurface, scanning near-field nanoscopy

## Abstract

Understanding the ultrafast excitation, detection, transportation, and manipulation of nanoscale spin dynamics in the terahertz (THz) frequency range is critical to developing spintronic THz optoelectronic nanodevices. However, the diffraction limitation of the sub-millimeter waves – THz wavelengths – has impaired experimental investigation of spintronic THz nano-emission. Here, we present an approach to studying laser THz emission nanoscopy from W|CoFeB|Pt metasurfaces with ∼60-nm lateral spatial resolution. When comparing with statistic near-field THz time-domain spectroscopy with and without the heterostructures on fused silica substrates, we find that polarization- and phase-sensitive THz emission nanoscopy is more sensitive than the statistic THz scattering intensity nanoscopy. Our approach opens explorations of nanoscale ultrafast THz spintronic dynamics in optically excited metasurfaces.

## 1 Introduction

When femtosecond laser pulses illuminate ferromagnetic films, ultrafast spin-polarized currents can be optically injected via efficient absorption [1], [2], [3], [4]. These longitudinal spin-polarized currents can be effectively converted to in-plane transverse charge currents when the ferromagnetic nanofilms are attached to heavy metals or topological insulators via the inverse spin Hall effect (ISHE). The in-plane transverse charge currents can coherently radiate THz electromagnetic waves by engineering the heterostructures of ferromagnetic metal and nonmagnetic material [5], [6], [7], [8], [9], [10]. Thanks to the highly efficient absorption of the ferromagnetic materials and laser THz emission experiments, the effective spin-to-charge conversion effect, spintronic THz emitters (STEs) with numerous advantages, such as high efficiency, ultra-broadband, ease for both integration and polarization manipulation, and low cost, have become ubiquitous [10], [11], [12], [13], [14]. STEs are adaptive for both femtosecond laser oscillators and amplifiers and do not have specific requirements for the pumping laser wavelengths. Consequently, STEs have been recognized as one of the most promising emitters for the next-generation THz sources [15]. Therefore, systematic investigations of the pump laser parameters, spintronic material types, and interface engineering have been conducted [16], [17], [18], [19], [20], [21], [22]. How to improve the radiated THz efficiency, manipulation of the bandwidth, and polarization have also been reported [23], [24], [25], [26], [27], [28]. Furthermore, strong-field THz emission has also been generated from STEs with a 4-inch huge size [29], [30]. Many experimental systems previously integrated with lithium niobate THz sources [23], [31] have been gradually equipped with STEs due to their versatile tunability [32]. The end faces of optical fibers can also be directly coated with such ferromagnetic metal|heavy metal heterostructure nanofilms. It makes the emission spatial resolution reach the micrometer scale. It may have the potential to replace commercial photoconductive antennas [33]. However, the development of functionalized STEs – integrating functions, such as beam splitting, focusing, waveform shaping, vortex beam generation, and so on – has been challenging to achieve experimentally due to the severe lack of accurate dielectric parameters of ferromagnetic metals, strict requirements for processing, and preparation conditions in THz frequency ranges for design and implementation. Therefore, twisting THz waves at the STEs is crucial for developing the next-generation high-performance functionalized THz sources.

Metamaterials/metasurfaces are artificial composite electromagnetic structures formed by subwavelength scale elements arranged in a certain macroscopic way, which can flexibly manipulate electromagnetic waves, and hence have also been employed a lot for THz beam focusing, splitting, and even for creating vortex THz beams [27], [34], [35], [36]. Most metamaterials/metasurfaces that used to work in the THz frequency range are made of gold materials [37]. However, the aforementioned functions are mostly separate from the sources. Is it possible to fabricate metamaterials/metasurfaces directly on the spintronic nanofilm heterostructures, thereby achieving high-efficiency functionalized STEs? There are still many difficulties and challenges. For example, can the properties of the ferromagnetic material itself be used as a material for the metasurfaces? Will multiple fabrication processes cause damage to the nanofilms? Will surface oxidation of materials lead to a decrease in radiation performance? Although recent work has shown that the above idea is feasible, studying the influences of the accurate fabrication processes down to nanoscale spatial resolution on the THz emission performance is highly demanded to further improve such device functions and qualities.

Laser THz emission microscopy (LTEM) [38] has been widely employed to probe micrometer-scale THz emission properties by tightly focusing the pumping laser via micro-objective lenses [23], [24], [25], [26]. Borrowing the merits of high spatial resolution from atomic force microscopy (AFM) and combining it with ultrafast THz time-domain spectroscopy to obtain nanoscale THz emission has become one of the best approaches [39–47]. Besides, advancing the static THz time-domain spectroscopy to nanoscale spatial resolution can also reflect the dielectric responses, while the far-field method can only give the average information in millimeter scales [48–53]. However, such powerful and versatile tools have not yet been applied to investigating STEs, especially spintronic THz metasurface emitters (STMEs) [32].

This work systematically characterizes STMEs via ultrafast THz scattering scanning near-field optical microscopy (THz s-SNOM) with a lateral spatial resolution of ∼60 nm [54]. When comparing the static THz wave scattering signals between W|CoFeB|Pt [18] and gold nanofilms, half of the scattering intensity of that from gold nanofilms is probed, implying the possibility of resolving the surface uniformity of the spintronic nanofilms. However, with the static THz near-field scattering signals from the STME nanofilms, it is relatively difficult to distinguish whether the W or Pt layers are on top of the trilayer samples on the substrates. Such obstacles can be overcome by employing laser THz emission nanoscopy (LTEN) by directly observing the THz emission signal amplitudes and phases. Through systematically studying the ultrafast dynamics process of the spintronic heterostructures under laser pumping and the THz emission performance, not only the ultrafast injection, detection, and control of the THz spin currents at the nanoscale are realized but also a new way to detect the flatness and oxidation degree of the sample surface is provided. It offers a reliable criterion preparing STMEs with higher performances and has an extremely important significance for interdisciplinary research in THz spintronics.

## 2 Experimental setup and sample preparation

**Figure 1:**
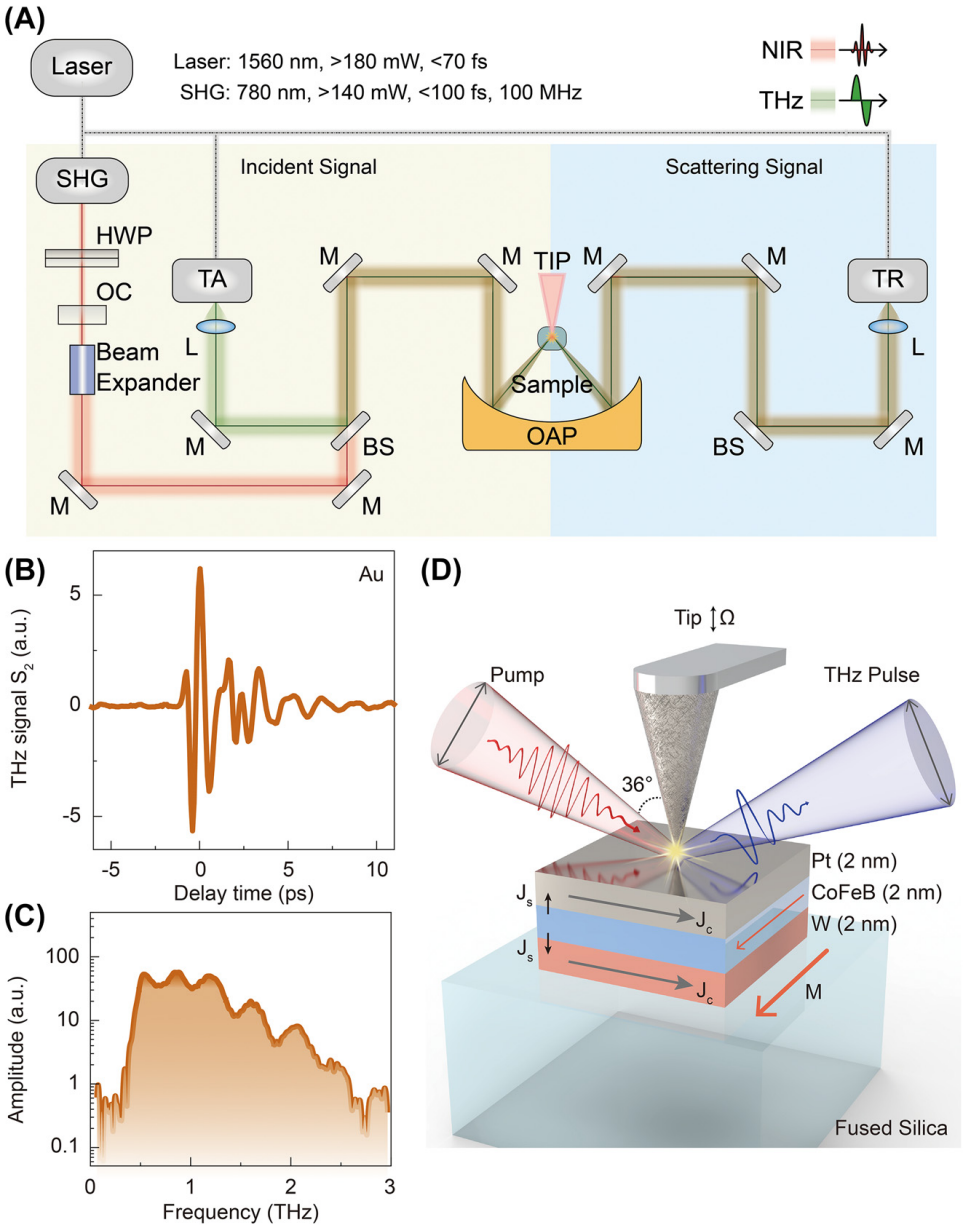
Schematic diagram of the experimental setup and its working principle. (A) Schematic of the THz s-SNOM system. SHG, second harmonic generation; HWP, half-wave plate; OC, optical isolator; M, mirror; TA, transmitter antenna; BS, beam splitter; OAP, off-axis parabolic mirror; RA, receiver antenna; L, lens. (B) A typical scattering near-field THz time-domain waveform and (C) its corresponding spectrum from a pure Au nanofilm on a fused silica substrate. (D) Schematic of the W|CoFeB|Pt STE.

To further improve the efficiency of the STEs and understand the generation, detection, and manipulation mechanism of the femtosecond spin currents at the nanoscale, we reduce the research scale from the far-field millimeter scale to the near-field nanoscale and observe the experimental results of the interaction between the femtosecond laser pulses and the heterostructure nanofilms. These experiments are implemented on an ultrafast THz s-SNOM (NeaSpec, Germany) to achieve the nanoscale spatial resolution. Figure 1(A) presents the overall optical path diagram of the ultrafast THz s-SNOM driven by a femtosecond fiber laser with a center wavelength of 1560 nm, a pulse width of 70 fs, and a repetition rate of 100 MHz. The femtosecond laser pulses are divided into three beams: one to trigger a second harmonic generator and two for THz photoconductive antennas. The former provides a free-space laser beam with a central wavelength of 780 nm for the optical pump–THz probe (OPTP) spectroscopy and LTEN. The other two paths are the excitation and the fiber-coupled THz time-domain spectrometer detection.

The THz pulses for the static near-field THz time-domain spectroscopy measurement are generated from the photoconductive InGaAs transmitter antenna (TA), pass through several THz reflecting mirrors and parabolic mirrors, and then are focused onto the sample surface via coupling with an AFM tip in the near-field. The THz scattering signal then passes through a series of symmetrical optical elements to the receiver antenna (RA) for ultrafast time-resolved detection. When the pumping laser is blocked, and the TA and RA are turned on, the system is in static THz time-domain spectroscopy measurement mode. In this case, the OPTP function can be realized by turning on the free-space modulating laser with a 780 nm central wavelength. In the OPTP mode, the THz emission signal can be observed by turning off the TA but keeping the RA in its working state. By coupling ultrafast laser pulses onto the AFM tips, the spatial resolution of the target materials and structures is no longer limited by the wavelength diffraction limit of the pumping laser. The tip radius predominantly determines it, and information on the spatial resolution of the tested samples at the nanometer scale can be obtained.

To calibrate the system, we first place a standard gold nanofilm with a thickness of 100 nm onto the sample holder. We purge the system with dry nitrogen gas. When the relative humidity go down to ∼4 %, we begin to extract the second-order static THz time-domain scattered signal from Au nanofilms, as shown in Figure 1(B). A typical single-cycle scattering temporal waveform is detected with a dynamic range of 1200, indicating that this system has a high scattering signal-to-noise ratio. Its corresponding spectrum is illustrated in Figure 1(C), manifesting that the near-field THz scattering spectral width can cover the range of 0.1–2.5 THz. The second-order signal localized through the tip is submerged in the far-field signal. However, we can restrict the far-field signal to the 0-order and 1-order signals using the tip resonance. Hence, the higher-order scattering signals obtained by demodulation have purer near-field information. Under this circumstance, we employ a modulation frequency of 48 kHz.

The STEs employed in this work were grown in a high-vacuum AJA sputtering system, and the substrate employed was fused silica. During the deposition, a sample rotation was performed to ensure good uniformity. In our case, each of the capping layers of W and Pt and the ferromagnetic layer of CoFeB are all 2 nm; thus, the total thickness of the trilayer sample is 6 nm. The samples were mounted on a custom-designed rotator with a fixed external static magnetic field (∼50 mT).

The main functions of ultrafast THz s-SNOM are OPTP and LTEN. In this work, as shown in Figure 1(D), the pumping laser pulse is first obliquely incident onto the AFM tip at an incidence angle of 36°. Through the near-field enhancement effect of the tip, a very high local-field enhancement can be obtained between the tip and the sample surface, and the CoFeB layer absorbs the localized laser energy. Due to the differences in the density and mobility of spin-up and spin-down electrons in the CoFeB layer, spin-polarized currents are generated in the longitudinal direction perpendicular to the sample surface. When these spin polarization currents move forward to the W layer and backward to the Pt layer, in-plane transversal charge currents are formed through the inverse spin Hall effect. Because W and Pt materials have intrinsically inverse spin Hall angles, and the longitudinal spin currents moving forward and backward in the W and Pt layers have opposite motion directions, the in-plane charge currents generated in both W and Pt have the exact directions. These charge currents carrying femtosecond time envelopes can coherently emit THz pulses according to the formula of $\vec{E_{th}}\propto \gamma \times \vec{{\text{j}}_{s}}\times \frac{\left|\vec{M}\right|}{{\vec{M}}_{s}}$ . Here, *γ* denotes the inverse spin Hall angle of W or Pt, and $\vec{M}$ represents the magnetization.

## 3 Scattering near-field THz spectroscopy from STEs

**Figure 2:**
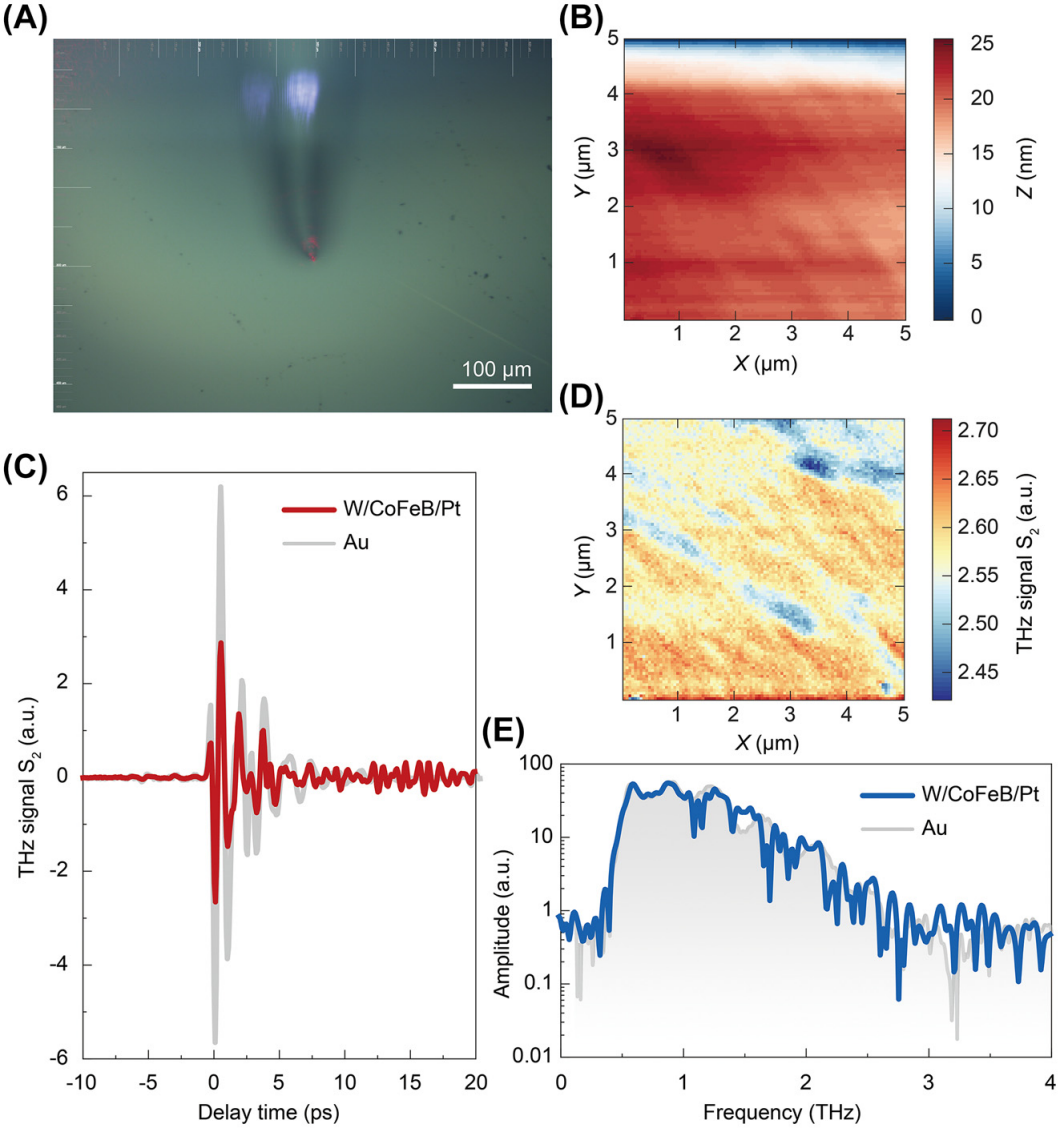
Comparison of the THz scattering signals between pure Au nanofilms and the W|CoFeB|Pt sample. (A) An optical photo of the STE surface with the cantilever. (B) AFM height topography. (C) Statistic THz time-domain scattering signal from gold nanofilms (100-nm thickness) and W|CoFeB|Pt (total thickness of 6 nm). (D) Mapping of the static THz scattering time-domain signal of the STE surface. (E) THz scattering spectra from the Au nanofilm and STE.

To characterize the scattering amplitudes and measurement capability, we first compare the THz scattering signals between pure gold nanofilms and W|CoFeB|Pt heterostructures. Figure 2(A) illustrates the optical photo of the W|CoFeB|Pt heterostructure sample surface and the tip cantilever. The size of this image is approximately 500 μm × 650 μm. The optical image shows that the sample surface is relatively flat, with a few defects or dust in some areas. Figure 2(B) depicts its corresponding height topography within a 5 μm × 5 μm area. The height difference is only up to 25 nm, indicating good flatness. Figure 2(C) depicts the static scattering THz time-domain waveforms measured from the W|CoFeB|Pt and gold nanofilm, respectively. Compared with that of the gold nanofilm, the scattering amplitude signal of the STE is half of it. The reason can be attributed to the total thickness of the STE being only 6 nm, and the THz wave can penetrate such thin nanofilms onto the fused silica substrate, experiencing a relatively weak reflection. The thickness of the gold nanofilm is 100 nm, which is already thick enough to reflect the incident THz waves fully. However, when fixing the signal extraction time at the peak of the THz temporal waveform in Figure 2(C) and mapping the STE sample surface, we can obtain THz scattering imaging concerning the area in Figure 2(B). The primary trend is consistent with AFM morphology; THz scattering spectroscopy is slightly uneven. This result manifests that the THz scattering nanoscopy is more sensitive than that from AFM morphology. Figure 2(E) exhibits the THz spectra for the STE and gold nanofilm, implying that the STE sample has a similar scattering spectrum.

**Figure 3:**
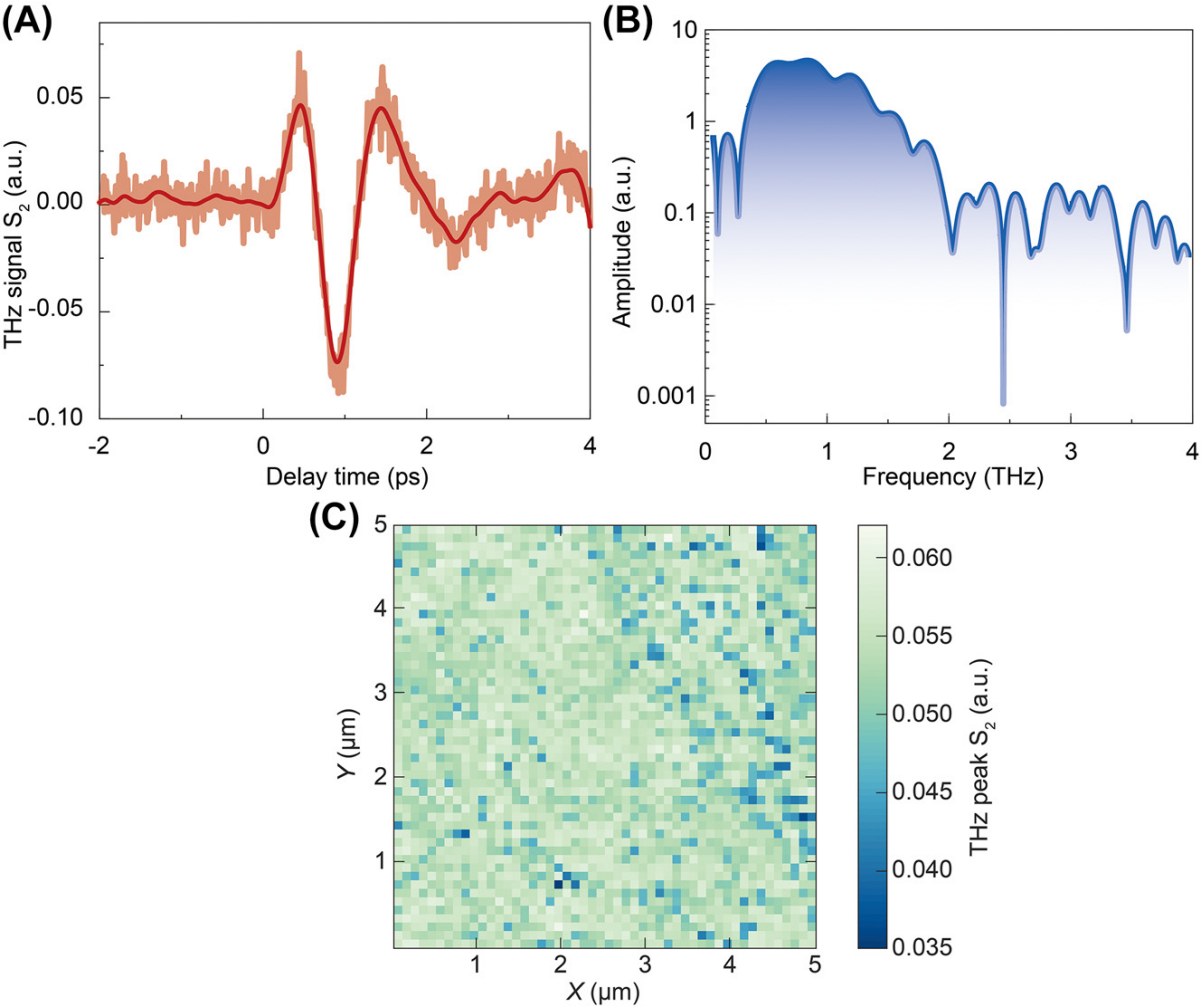
THz emission nanoscopy from an STE without artificial structures. (A) Second-order THz emission time-domain waveform and (B) the corresponding spectrum. (C) STEN image of flat sample.

We carried out laser-pump THz emission experiments on the THz s-SNOM system to examine the STE performance further. It can be seen from the time-domain waveform in Figure 3(A) that the THz radiation produced by STE is a single-cycle pulse with a pulse width of ∼1 ps. Figure 3(B) shows the corresponding spectrum with a THz peak frequency of 1 THz and a spectral width of 2.0 THz. In the case of purging with nitrogen gas in the setup, the absorption peak of water vapor can be well eliminated. On this basis, we map the THz emission temporal waveform intensity, and the signal shows no apparent differences, implying that the specific area had good flatness without any artificial structures.

## 4 Near-field emission and imaging of spintronic THz metasurfaces

Metasurface samples based on STEs have been demonstrated with versatile modulation properties in the far field [27]. By varying the stacking order of the STE nanofilms, the direction of the spin currents in the nanofilms can be fully reversed, which further prepares the phase of the emitted THz pulse to have a 180° phase difference. Considering this, adjusting the THz emission angle and focusing status is possible by appropriately designing metasurface patterns. Here, we conducted experiments on focused ring samples, characterized and analyzed by studying their AFM height topography, THz scattering signal intensity mapping, and emission performance on the nanometer scale.

**Figure 4:**
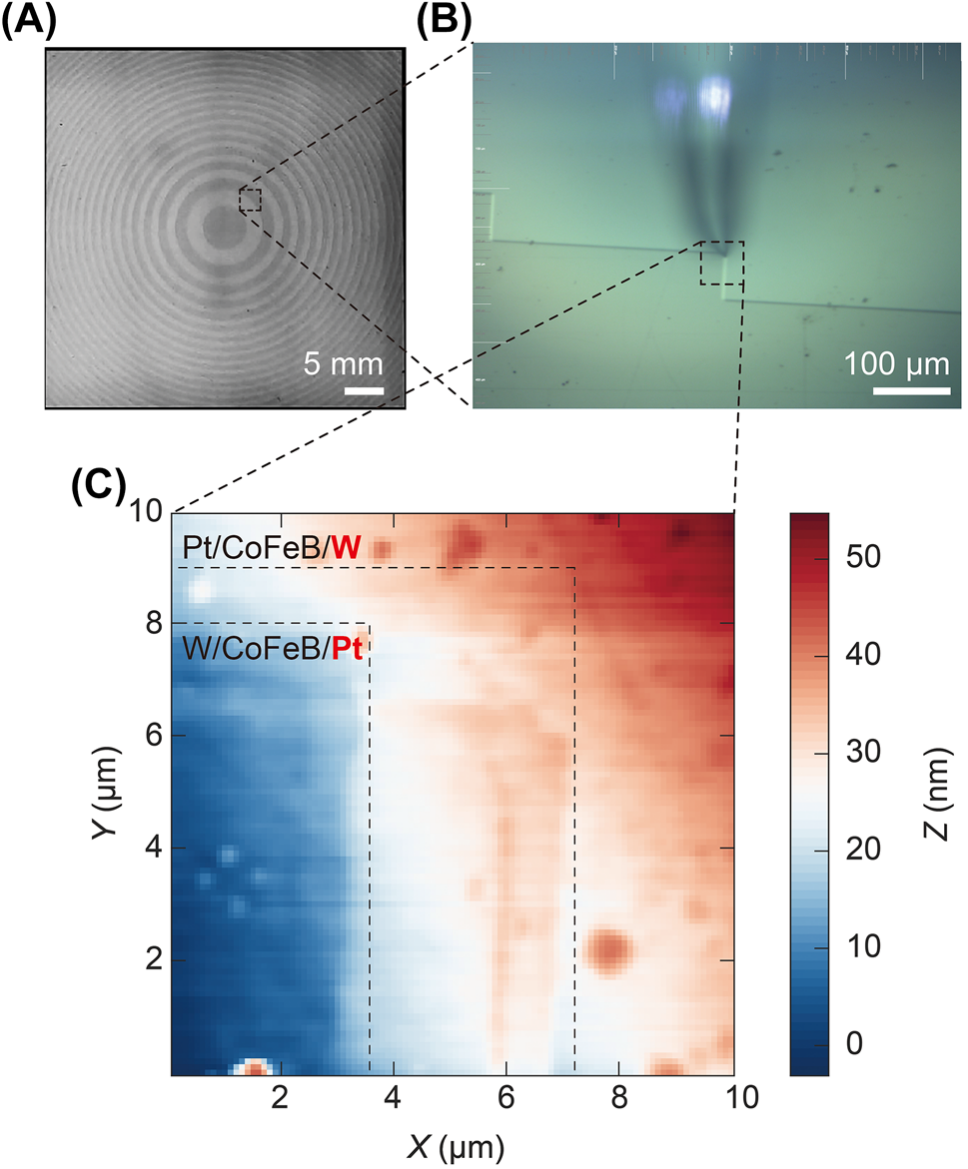
AFM morphology characterization of the STME sample surface. (A) Optical photograph of the STME sample surface, where the scale bar is 5 mm. (B) An enlarged area imaged under an optical microscope, where the scale bar is 100 μm. (C) Zoomed-in AFM surface height topography.

Figure 4 illustrates the sample surface information of the STME sample. Figure 4(A) shows the complete optical photo of the sample, and the scale bar is marked as 5 mm. This figure shows that the sample surface is arranged as a ring metasurface structure, where the dark area is Pt|CoFeB|W with Pt on the top of the trilayer sample. In contrast, the light color structure is W|CoFeB|Pt with W on the top. The reason for this result is that W is more easily oxidized. When there is no capping layer, it will change to a light color due to oxidation. The gray curve area in the figure is selected and placed under an optical microscope for further characterization. We can see more detailed sample surface information in Figure 4(B). Macroscopically, the structure appears as a ring. However, it presents a zigzag shape at the edges when enlarged to a micrometer size. We select one of the sample boundary areas for magnification, and the obtained AFM morphology is plotted in Figure 4(C). Due to the irregularity of the sample itself, we can see several noticeable bulges in the results, which may be the defect left by sample preparation or the dust falling on the sample surface. The result of Figure 4(C) can be divided into three regions, with the blue region with the most negligible thickness being the Pt layer on top, the red region with the most significant thickness being the W layer on top, and the white region in the middle being the part where the two layers stack. The experimental results indicate that the W layer is thicker than the Pt layer due to the occurrence of the oxidation phenomenon. However, the sample is not newly processed and has been stored in the laboratory for some time.

**Figure 5:**
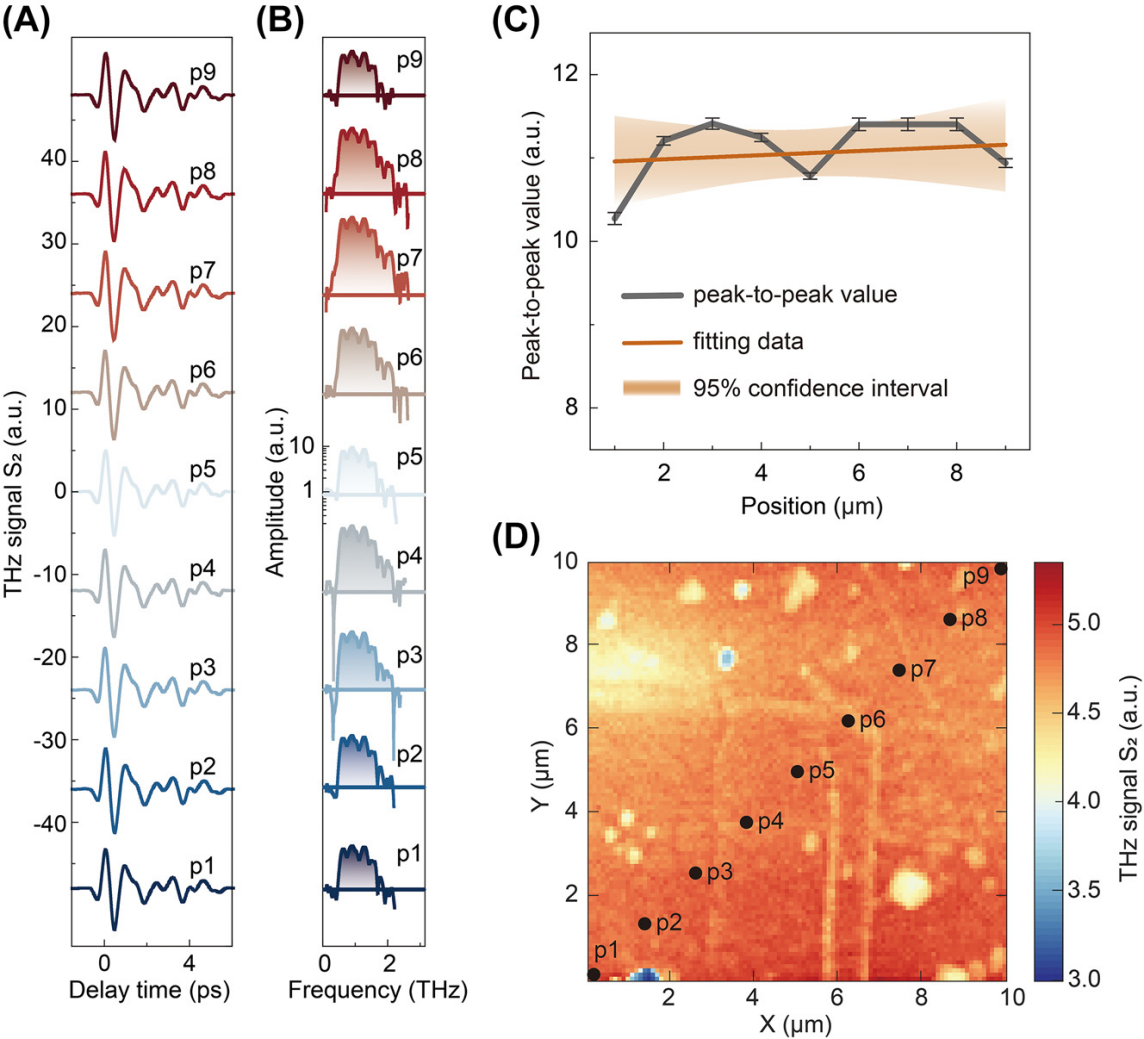
Statistic THz scattering intensity mapping of STME. (A) THz scattering time-domain waveform and (B) the corresponding spectrum. (C) Peak-to-peak value and signal fitting of THz scattering signals at different positions. (D) THz scattering intensity map of the same region is shown in Figure 4(C).

To further explore the feasibility of using static THz time-domain nanoscopy to distinguish W|CoFeB|Pt and Pt|CoFeB|W on this sample, we employ the THz s-SNOM to characterize the STME edges with the same zoomed-in area in Figure 4. The resulting THz scattering temporal waveform intensity mapping of the STME sample surface is depicted in Figure 5(D). This result is consistent with the AFM in Figure 4(C). There may appear to be some subtle signal differences at the edges of the metasurface. We select different positions of p1–9 to test the complete time waveforms and their corresponding spectra, as shown in Figure 5(A) and (B). Furthermore, we extract the peak-to-peak values of the THz scattering signals at these nine different positions and fit them to obtain the results shown in Figure 5(C). After fitting, it is found that the peak-to-peak value of the signal does not change much with the change of position, which is consistent with the THz scattering intensity graph shown in Figure 5(D). This result indicates that traditional THz scattering measurements cannot distinguish the structure of such metasurface materials.

**Figure 6:**
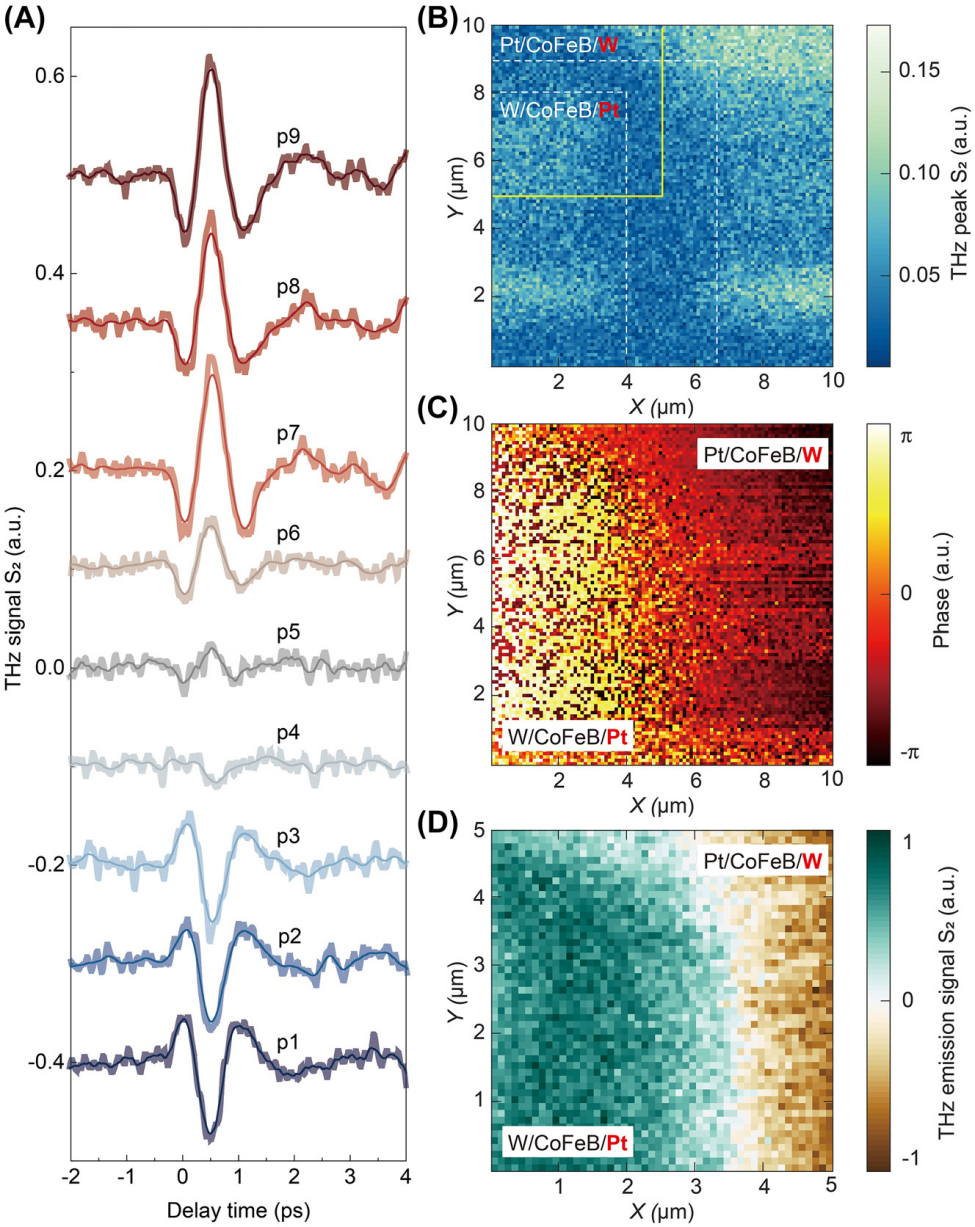
THz emission nanoscopy mapping of the STME sample surface. (A) THz time-domain waveforms at different positions. (B) THz emission amplitude mapping and its (C) corresponding phase spectrum. (D) Normalized THz emission signal in the yellow area indicated in (B).

Up to now, it has been demonstrated that no matter the AFM tomography or the statistic THz scattering intensity mapping methods, it is very challenging to distinguish the W|CoFeB|Pt-substrate and Pt|CoFeB|W-substrate STME samples, needless to say, the oxidization status for W layer. However, LTEN may have more chances. As shown in Figure 6, we conduct the THz emission measurement and obtain the THz emission signals at p1–9, summarize and plot in Figure 6(A). It is evident that in this metasurface material, due to the inconsistent stacking order of the spintronic emission film sample, different emission signals with polarity reversal phenomenon are obtained at different Pt|CoFeB|W and W|CoFeB|Pt samples. Positions p1–3 represent the emission signal of the spintronic film sample with the Pt layer on top. As the position changes from p1 to p3, it becomes closer to the edge of this part of the sample, resulting in a decrease in sample purity and a gradual weakening of the emission signal. As the position changes, the Pt and W layers are mixed on the upper sample in the p4–5 region due to the degree of processing precision. Both positive and negative signals result in the overall absence of weak THz emission characteristics in this region. As the position moves from p7 to p9, the sample again becomes a pure W layer on top of the sample. The THz emission signal has an opposite phase, and its intensity becomes stronger. Moreover, as the purity of the sample gradually improves, the signal quality also gradually improves.

Next, we conduct a THz emission amplitude scan in this area, and the results obtained are shown in Figure 6(B). Due to software limitations, we directly measure the signal’s amplitude, which is all positive. As for samples with negative electric fields, we cannot see significant signal differences and can only distinguish areas where there is no obvious emission signal at the overlapping positions of the samples. Although current experimental results indicate that strength information can somewhat be distinguished, this may become an essential method for characterizing metasurface materials. However, at this stage, we have found that the phase information spectrum shown in Figure 6(C) can intuitively display the flipping of this signal. To extract the accurate THz emission information, we selected the area within the yellow box in Figure 6(B) to scan the complete time-domain waveform at each pump delay time. We obtain their actual peak values by processing this massive amount of data and plot the normalized data in Figure 6(D). Here, we can see the W/CoFeB/Pt regions marked in brown, while the Pt/CoFeB/W regions in green are easily discriminated. These results show that spintronic THz emission nanoscopy is a reliable and powerful tool for characterizing STMEs.

## 5 Conclusions

In summary, this work presents the nanometer scale characterization of STE and STME samples employing an ultrafast THz s-SNOM system. By comparing the optical microscopy, AFM height topography, and THz scattering intensity spectrum, it is found that the surface THz scattering signal is the basis for material characterization in studying STE materials. At the same time, in the metasurface structure based on the STE surface, the emergence of the THz emission nanoscopy provides a new phase-sensitive method for characterizing the sample surface, including surface oxidization. Based on these observations, we can achieve more accurate detection and characterization tools for the STEs, providing a foundation for the fabrication and production of high-performance THz emitters with versatile integrated functions. Our results will not only promote the laser terahertz emission nanoscopy to the nanometer scale but also realize the possibility of applying THz s-SNOM to the study of nanometer scale ultrafast magnetism and ultrafast THz spintronic optoelectronics, which will have a specific role in promoting nano-spintronics, ultrafast nano-photonics, THz nanotechnology, and their interdisciplinary disciplines.
